# Inhibition of FOSL2 aggravates the apoptosis of ovarian cancer cells by promoting the formation of inflammasomes

**DOI:** 10.1007/s13258-021-01152-6

**Published:** 2021-11-13

**Authors:** Jie Li, Li Zhou, Hongye Jiang, Lin Lin, Yinguang Li

**Affiliations:** 1 Department of Gynecology, The Sixth Affiliated Hospital, Sun Yat-Sen University, No. 26, Yuancun Erheng Road, TianHe District, Guangzhou, 510655 China; 2 Department of Gynecology, The First Affiliated Hospital of Sun Yat-Sen University, Guangzhou, China

**Keywords:** FOSL2, Inflammasome, Ovarian cancer, Yvad-CMK

## Abstract

**Background:**

Ovarian cancer is a common gynecological malignancy among female patients and poses a serious threat to women’s health. Although it has been established that Fos-like antigen 2 (FOSL2) is linked to ovarian cancer (OC), its exact role in the development of OC remains unknown.

**Objective:**

This article aims to investigate the role of FOSL2 in ovarian cancer development.

**Methods:**

FOSL2 expression in ovarian carcinoma and adjacent tissues was assessed using real-time fluorescent quantitative PCR and western blot. We constructed OE/sh-FOSL2 plasmids and Caspase-1 specific inhibitors (Yvad-CMK) and transfected A 2780 cells with them to identify the relevant cell functions. Furthermore, we used western blot assay to determine the changes in expression of apoptosis-associated speck-like protein containing a CARD (ASC), cysteine aspartate-specific proteasezymogen procaspase 1 (pro-caspase-1), cysteinyl aspartate-specific proteinase-1 (caspase-1), interleukin-1β precursor (pro-IL-1β), interleukin-1β (IL-1β), interleukin-18 precursor (pro-IL-18), and interleukin-18 (IL-18). In addition, we measured the concentration of IL-1β and IL-18 using an enzyme-linked immunosorbent assay (ELISA). Moreover, Tthe level of lactate dehydrogenase (LDH) in the cell supernatant was measured by LDH release assay kit.

**Results:**

The expression of FOSL2 was significantly higher compared with the surrounding tissues. The proliferation, migration, and invasion of A2780 cells were enhanced after transfection with OE-FOSL2 plasmids; however, the cell apoptosis was significantly decreased. When FOSL2 was overexpressed, the inflammasome-associated proteins such as ASC, caspase-1, IL-1β, and IL-18 were downregulated. Furthermore, FOSL2 induced apoptosis and activated the production of inflammasomes in A2780 cells. Co-therapy with Yvad-CMK and substantially inhibited apoptosis and activation of inflammasomes.

**Conclusions:**

Inhibition of FOSL2 promotes the apoptosis of OC cells by mediating the formation of an inflammasome.

## Introduction

Ovarian cancer (OC) is a prevalent gynecological malignancy among women (Stewart and Lockwood 2019). It has the highest morbidity among malignant genital tumors and the highest mortality rate among gynecologic tumors, making it a serious threat to women’s health (Kossaï et al. 2018; Menon and Gentry-Maharaj 2018). Despite recent advances in the treatment of OC, the majority of patients are diagnosed in the advanced stage (Sehouli 2019). Therefore, identifying the relevant molecular mechanism is important for the diagnosis and treatment of OC.

Fos-like antigen 2 (FOSL2) is a member of the activator protein-1 (AP1) transcription family that is widely expressed in various tissues and regulates the growth, development, reproduction, immune response, and other biological functions (Li et al. 2018). Although several studies have demonstrated that FOSL2 mediates the progression of colon cancer, hepatocellular carcinoma (HCC), and other malignancies by regulating the epithelial-mesenchymal transitions (EMT), metastasis, and other malignant behaviors (Chen et al. 2020; Li et al. 2018, 2020), little is known about the role of FOSL2 in the occurrence and progression of OC.

Inflammasomes are multi-protein complexes composed of pattern recognition receptors (PRRs), apoptosis-associated speck-like protein containing a CARD (ASC), and an effector molecule cysteine-dependent aspartate hydrolase-1 (pro-caspase-1). When eukaryotic cells detect the pathogen-associated molecular pattern (PAMP) derived from infected pathogens and the dang-associated molecular pattern (DAMP) derived from damaged host cell cytoplasm, NLRP3 undergoes self-oligomerization through conformational changes and recruits the junction protein ASC through PYD–PYD interaction. ASC then recruits them to the NLRP3-ASC complex by binding to the caspase-recruitment domain of pro-caspase-1, thus completing the assembly of the NLRP3 inflammasome (Chen et al. 2017; Malik 2017; Mezzasoma and Talesa 2017). Furthermore, this complex converts Procaspase-1 into enzyme-active Caspase-1, which further generates pro-inflammatory cytokines interleukin-1β precursor (pro-IL-1β) and interleukin-18 precursor (pro-IL-18) and promotes their maturation (Ding et al. 2016).

Cysteinyl aspartate-specific proteinase 1 (caspase-1) is a member of the inflammasome-associated caspase family. Several studies have shown that tumor cells show a decreased caspase-1 expression, and over-expression of caspase-1 can inhibit the growth of tumor cells (Bo Hu et al. 2010; Lasithiotaki et al. 2018). Qiang Feng et al. (2005) showed that the tumor growth in mice with caspase-1 deficiency was considerably enhanced, and these mice exhibited increased colon epithelial cell proliferation in the early stage and attenuated cell apoptosis in the late stage (Qiang Feng et al. 2005). Interestingly, similar phenomena were observed in both prostate and OCs, suggesting that targeting caspase-1 may be a new approach to tumor therapy (Chang et al. 2017; Feng et al. 2005; Winter et al. 2001). However, it is unclear whether caspase-1 regulates the progression of OC through the formation of inflammasome and if FOSL2 regulates this process.

This study demonstrated that FOSL2 expression was greatly upregulated in OC tissues compared with the neighboring tissues. Moreover, downregulation of FOSL2 suppressed proliferation, migration, and invasion of A2780 cells and induced the formation of inflammasomes. These inflammasomes further promoted cell apoptosis. However, these phenomena were reversed when tissues were treated with Yvad-CMK. Therefore, our findings revealed that FOSL2 knockdown upregulated the expression of ASC, which was further bound to the caspase-recruitment domain of pro-caspase-1 and promoted the formation of inflammasomes. This increased the expression of caspase-1, IL-1β, and IL-18, promoting apoptosis and suppressing proliferation, migration, and invasion of OC cells. Our research provided evidence that FOSL2 regulates the malignant biological behaviors of OC cells by mediating the formation of inflammasomes. Therefore, FOSL2 could be used as a novel therapeutic target for the treatment of OC.

## Material and methods

### Collection of clinical samples

We collected 20 OC and their matched peritumor tissue samples from patients who underwent radical resections at The Sixth Affiliated Hospital of Sun Yat-sen University (Guangzhou, China). We have obtained informed consent from each patient before collecting samples. This study was approved by The Sixth Affiliated Hospital of Sun Yat-sen University Institutional Review Board.

### Cell culture and treatment

Human OC cells A2780 were purchased from ATCC (Manassas, VA, USA) and cultured at 37 °C with 5% CO_2_ in RPMI 1640 medium (Hyclone, Logan, UT, USA) containing 10% fetal bovine serum (Hyclone). To evaluate the effect of FOSL2 on OC cells, the A2780 cells were treated with either pcDNA4.0-FOSL2 plasmid or FOSL2 shRNA. The cells were randomly allocated into five groups: control, pcDNA4.0 (OE-NC), pcDNA4.0-FOSL2 (OE-FOSL2), control shRNA (shCTRL), and FOSL2 shRNA (shFOSL2). After 48 h, the OC cell function and the expression of inflammasome-associated proteins were assessed to determine the effect of FOSL2 on the formation of inflammasome and the apoptosis of OC cells. Furthermore, to confirm the role of inflammasomes in inhibiting OC cell apoptosis, Ac-Yvad-CMK was used to block inflammasome activation. For this experiment, cells were randomly divided into four groups: control, control shRNA (shCTRL), FOSL2 shRNA (shFOSL2), and FOSL2 shRNA + Yvad-CMK (400,012, Sigma, 20 μM) (shFOSL2 + Yvad-CMK).

### Cell transfection

Using lipofectamine2000 (Invitrogen, USA), A2780 cells were transfected with either pcDNA4.0-FOSL2 (50 nM) plasmid or FOSL2 shRNA (50 nM) and then selected with neomycin (800 μg/ml) for 4 weeks. pcDNA4.0 and control shRNA were negative controls. We acquired all the plasmids and shRNAs from Ribobio (Guangzhou, China).

### Real-time PCR

Total RNAs were extracted from cells using Trizol reagent (Takara, Dalian, China). The first-strand cDNA was generated using the Reverse Transcription System Kit (Takara) and amplified and detected using the SYBR Green kits (Takara) on the Applied Biosystems’ 7500 StepOne Plus system (Applied Biosystems, CA, USA). We used the following primer sequences: FOSL2 forward 5′- CAGAAATTCCGGGTAGATATGCC-3′, reverse 5′- GGTATGGGTTGGACATGGAGG-3′; GAPDH forward 5′- TGTTCGTCATGGGTGTGAAC -3′, reverse 5′- ATGGCATGGACTGTGGTCAT -3′. Furthermore, the relative gene expressions were calculated by 2^−△△ct^ method.

### Western blotting

5 × 10^6^ cells at the logarithmic growth stage were collected. To extract the total proteins, RAPI lysate (Beyotime, Nanjing, China) containing protease and phosphatase inhibitors was used. The relevant protein concentration was measured using a BCA kit (Thermo Fisher Scientific, MA, USA). After quantification, the proteins were separated using SDS-PAGE. These proteins were transferred to polyvinylidene fluoride (PVDF) membranes and sealed in 5% degrease milk for 1 h. The blots were incubated at 4 ℃ overnight with the primary antibodies against ASC (CST, 67,824, 1:1000), pro-caspase-1 (Abcam, ab32499, 1:10,000), caspase-1 (Santa, sc-56036, 1:500), IL-1β (CST, 12,703, 1:1000), IL-18 (CST, 54,943, 1:1000), and GAPDH (Abcam, ab181602, 1:10,000). The membranes were washed and incubated with horseradish peroxidase (HRP)-conjugated antibodies at 23 °C for 1 h and then developed using ECL-Plus reagent (Thermo Fisher Scientific). We observed the membranes using gel imaging system and analyzed the findings.

### CCK-8 assay

One day before the experiment, we transfected A2780 cells with relevant plasmids and inoculated them in 96-well plates with 5 × 10^3^ cells per well. After 24, 48, and 72 h, respectively, the CCK-8 solution (10%, Dojindo, Japan) was added to each well and incubated at 37 °C for 2 h. The optical density was measured at 450 nm.

### 5-ethynyl-2′-deoxyuridine *(EdU) staining*

Transfected A2780 cells were incubated with 50 μM EdU (Solarbio, Beijing, China) for 2 h to label these cells with EdU. After staining nuclei of these cells with DAPI, we observed them under a fluorescence microscope (Olympus, Tokyo, Japan).

### TUNEL assay

After 48 h, A2780 cells were fixed with 4% paraformaldehyde (PFA) and then incubated for 60 min with TUNEL assay mixture (Roche, Mannheim, Germany; Cat#11684817910). Following staining cell nuclei with DAPI, these cells were observed under a fluorescence microscope (Olympus, Tokyo, Japan).

### Cell apoptosis assay

After transfection and treatment as described earlier, cells were collected and re-suspended in 400 μL labelled buffer. We added 5 μL Annexin V-FITC (Beyotime, China) and 10 μL propidium iodide (PI) to this suspension and incubated it for 20 min in dark conditions. The cell apoptosis was determined using a flow cytometer (BD Biosciences, San Jose, USA).

### Wound healing assay

We seeded transfected A2780 cells into 24-well plates (Corning, NY, USA) and cultured them to form a monolayer on the bottom of the plates. After culturing these cells overnight, we scratched the monolayer of each well in a straight line using 200 μL micropipette tips. The dropped cells were rinsed away and the adhering cells were cultured in the complete mediums. The wound width was measured using a microscope (Nikon, Japan) at 0, 24, and 48 h. We calculated the relative wound area using Image pro plus 6.0 (Media Cybernetics, USA) compared with 0 h.

### Invasion assay

First, the Matrigel matrix (BD Biosciences) was mixed with the RPMI-1640 medium. This mixture was uniformly distributed to the bottom of each well (80 μL/cell) and then solidified in the incubator. After solidification, 200 μL of RPMI-1640 medium containing 1 × 10^4^ cells was added to the upper chamber, and the medium containing 10% FBS (700 μL) was added to the lower chamber. After 48 h, we removed the matrix glue and fixed cells with 4% paraformaldehyde and dyed them with 0.4% crystal violet (Sigma-Aldrich). Cells from 10 fields were randomly selected to be photographed and counted under the light microscope, and the average value was recorded for statistical analysis.

### Detection of IL-1β, IL-18, and LDH

An ELISA test identified inflammatory cytokines IL-1β and IL-18 in the supernatant. Furthermore, the activity of LDH in the supernatant was determined using LDH release assay kit (MBS3801209). All ELISA kits were supplied by eBioscience (Thermo Fisher Scientific).

### Statistical analysis

We used GraphPad Prism 7.0 software (GraphPad, CA, USA) to process study data. These data were represented as mean ± standard deviation. To compare the two groups and multiple groups, unpaired *t*-tests and *one-way ANOVA* were used, respectively. Data considered statistically significant if the *P*-value was less than 0.05.

## Results

### FOSL2 was greatly increased in OC tissues

**Fig. 1:**
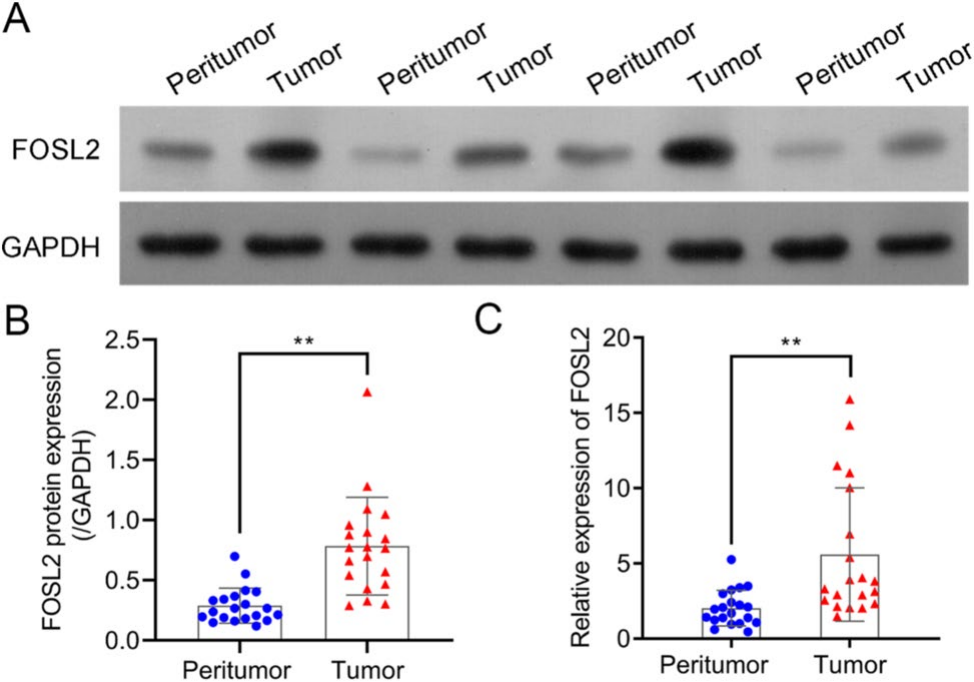
FOSL2 was greatly increased in clinical samples. FOSL2 expression in ovarian cancer tissues and their pair-matched peritumor tissues were detected through western blot (**A** and **B**) and real-time PCR (**C**), respectively. **P < 0.01 vs. peritumor tissues

FOSL2 expression was detected by qPCR and western blot analysis in OC tissues and their matched neighboring tissues. FOSL2 expression was higher in OC tissues than that in peritumor tissues (Fig. 1A–C).

### FOSL2 knockdown suppressed the proliferation, migration, and invasion of A2780 cells

**Fig. 2:**
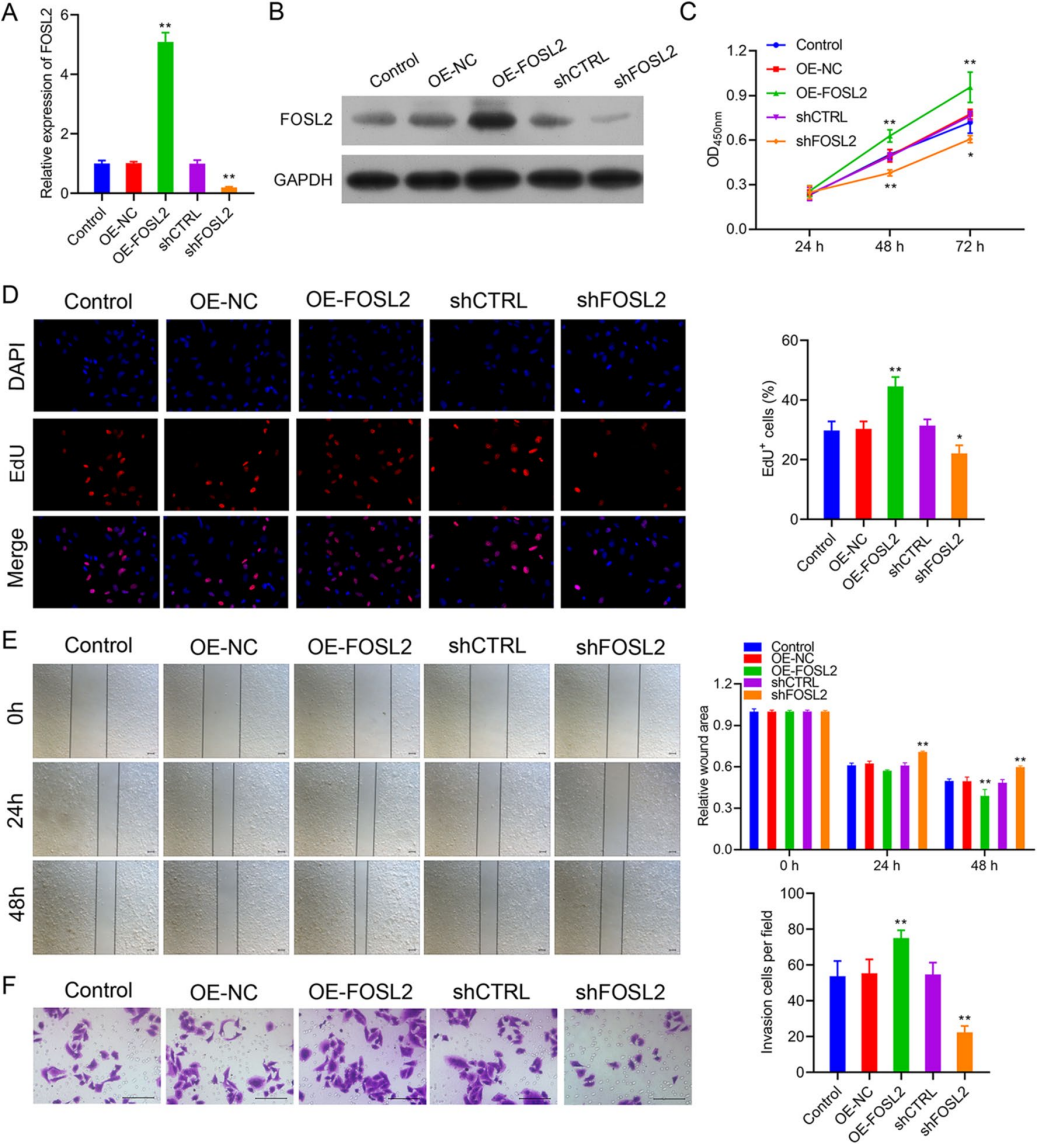
FOSL2 knockdown greatly suppressed the proliferation, migration and invasion in A2780 cells. The transfection efficiencies were observed by real-time PCR (**A**) and western blot (**B**). The effect of FOSL2 on cell proliferation were observed via CCK-8 assay (**C**) and EdU staining (**D**). The effect of FOSL2 on cell migration and invasion were observed by wound healing (**E**) and transwell assay (**F**). *P < 0.05, **P < 0.01 *vs*. control group

To explore the role of FOSL2 during OC development, the A2780 cells were transfected with OE-FOSL2 plasmid or FOSL2 shRNA, and the transfection efficiencies were confirmed using real-time PCR and western blot, respectively. The findings revealed that transfecting A2780 cells with OE-FOSL2 plasmid or sh-FOSL2 significantly increased or decreased the expression of FOSL2 (Fig. 2A,B). These results suggested that A2780 cells with stable overexpression or knockdown of FOSL2 may be used to explore the function of FOSL2 in OC progression. Furthermore, CCK-8 and EdU staining results demonstrated that overexpression of FOSL2 promoted cell proliferation, whereas FOSL2 knockdown inhibited this phenomenon (Fig. 2C, D). Moreover, we observed that overexpression of FOSL2 significantly enhanced cell migration and invasion in A2780 cells. On the other hand, after FOSL2 silencing, the wound healing and transwell assays produced opposite results (Fig. 2E, F). These findings revealed that overexpression of FOSL2 amplified the malignant biological behavior of A2780 cells, whereas the FOSL2 knockdown showed opposite effects.

### FOSL2 knockdown promoted the cell apoptosis and inflammasome activation

**Fig. 3:**
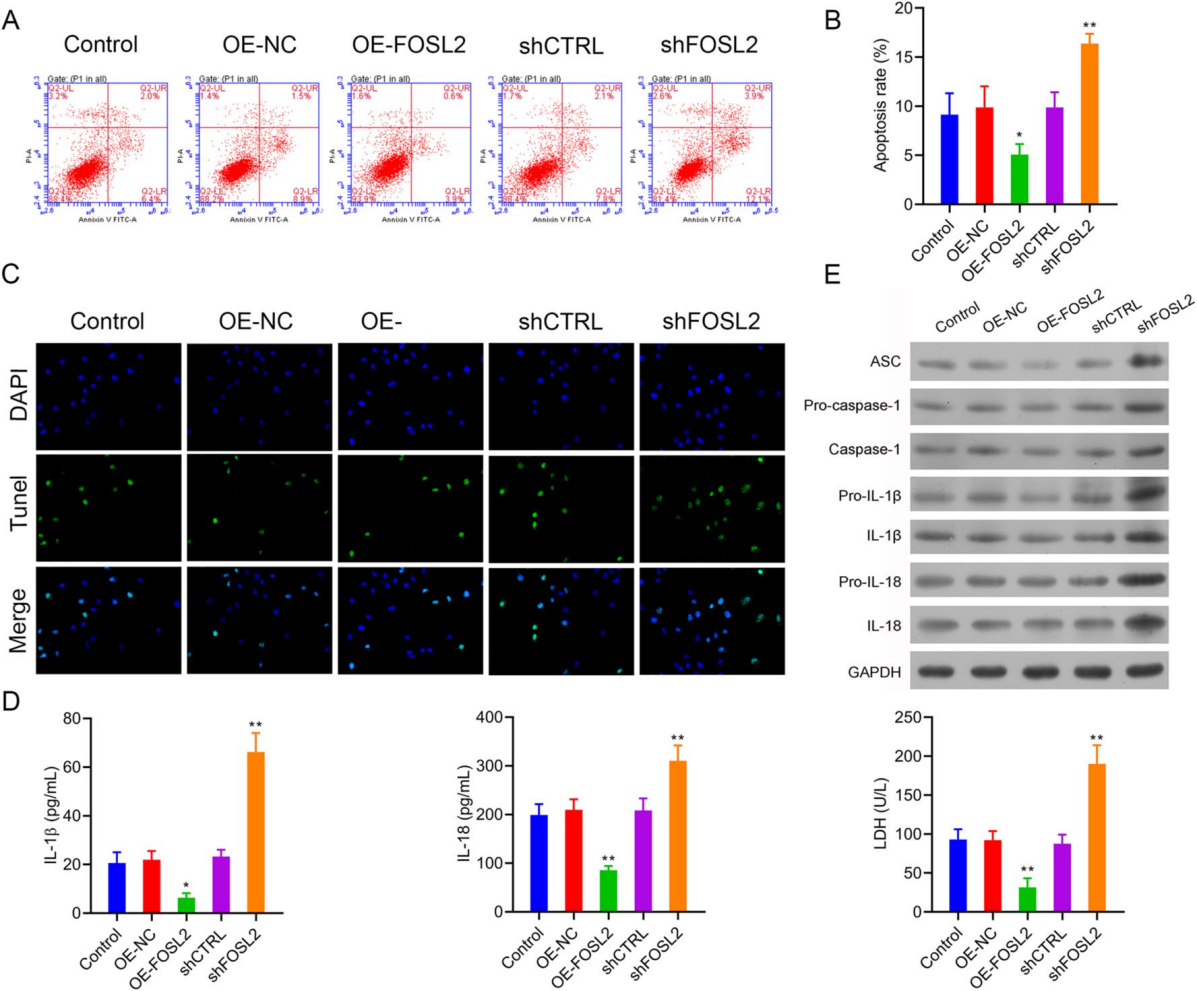
FOSL2 knockdown could promote the cell apoptosis and inflammasome activation. **A **and **B** The cell apoptosis was detected by flow cytometry and TUNEL staining (**C**). **D** The secretion of IL-1β and IL-18 were detected by ELISA assay, and the LDH activity in cell supernatant was detected by LDH release assay kit. **E** The expression of ASC, pro-caspase-1, caspase-1pro-IL-1β, IL-1β, pro-IL-18 and IL-18 were analyzed by western blot. *P < 0.05, **P < 0.01 *vs*. control group

The influence of FOSL2 on cell apoptosis was detected through flow cytometry and TUNEL staining. When compared with their control group, we found that overexpression of FOSL2 in A2780 cells significantly reduced cell apoptosis, whereas FOSL2 knockdown increased cell apoptosis (Fig. 3A–C). Furthermore, we evaluated the cell damage by measuring LDH activity in cell culture supernatant and found that the LDH activities were markedly decreased by overexpression of FOSL2 but significantly increased when FOSL2 was knocked down in A2780 cells (Fig. 3D, right panel). In addition, expression of proteins associated with inflammasomes formation, including ASC, pro-caspase-1, caspase-1, pro-IL-1β, IL-1β, pro-IL-18, and IL-18 expression was markedly upregulated when FOSL2 was knockdown (Fig. 3E). Similar findings were achieved for IL-1β and IL-18 identified in A2780 cells using ELISA (Fig. 3D, left and middle panel). All of these findings demonstrated that FOSL2 knockdown promotes cell apoptosis and inflammasomes activation.

### Yvad-CMK significantly reversed the effect of FOSL2 on cell proliferation, migration, and invasion

**Fig. 4:**
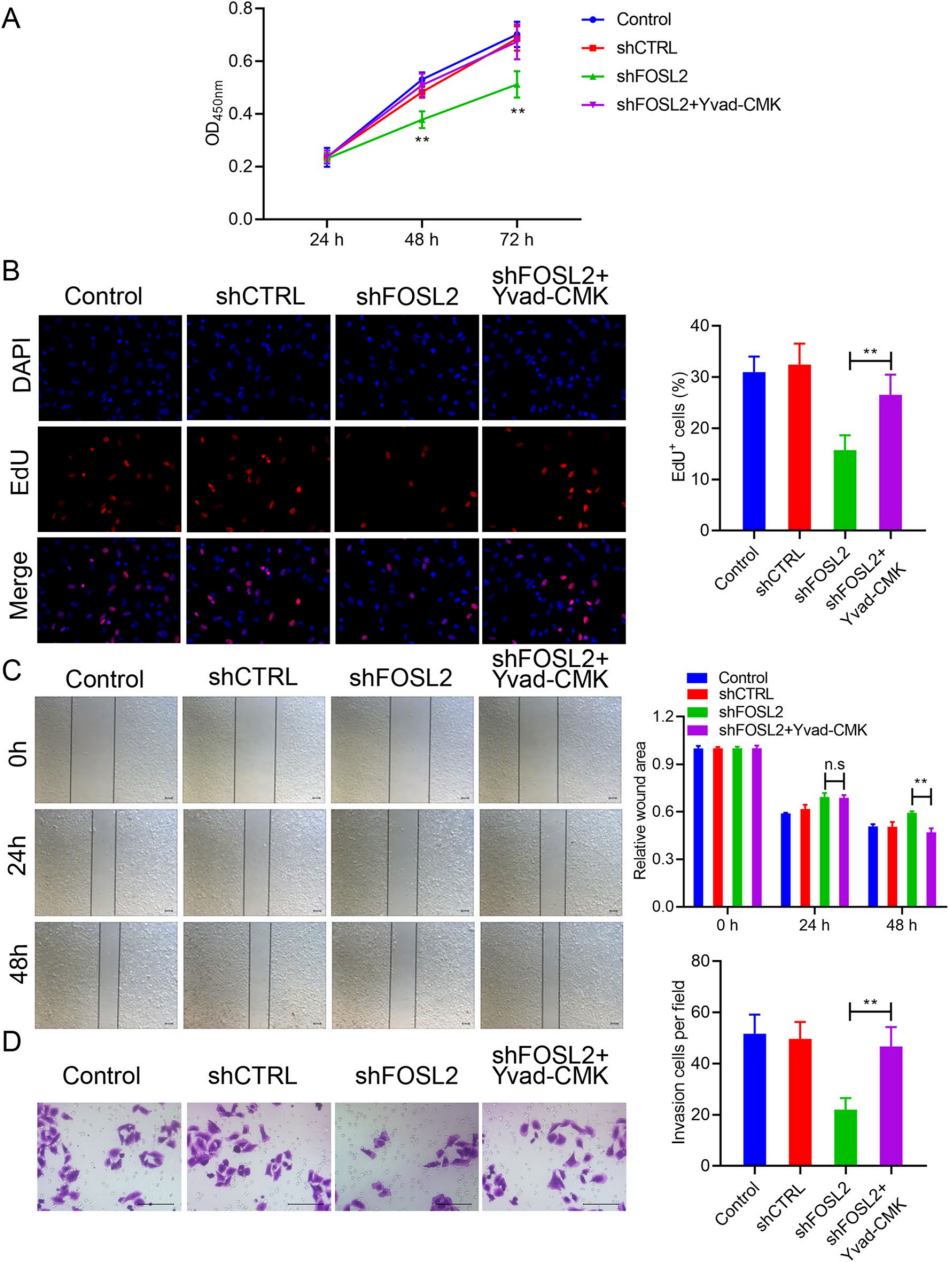
The effects of FOSL2 on cell proliferation, migration and invasion were markedly reversed by Yvad-CMK. The effect of FOSL2 and Yvad-CMK on cell proliferation was observed via CCK-8 assay (**A**) and EdU staining (**B**), respectively. The effect of FOSL2 and Yvad-CMK on cell migration and invasion were observed by wound healing (**C**) and transwell assay (**D**). **P < 0.01 *vs*. shFOSL2 group

Although knockdown of FOSL2 increased inflammasomes activation, the role of inflammasomes in the apoptosis of A2780 cells induced by FOSL2 knockdown remains unclear. In this study, we used Yvad-CMK to suppress the inflammasome activation in FOSL2 knockdown A2780 cells and then examined the effect on cell proliferation, migration, and invasion. The findings indicated that cell proliferation inhibited by FOSL2 knockdown was reversed by Yvad-CMK treatment (Fig. 4A, B). Moreover, Yvad-CMK treatment restored the migratory and invasive capacities reduced by FOSL2 knockdown (Fig. 4C, D). These results demonstrated that inhibiting the activation of the inflammasome significantly reverses the effect of FOSL2 knockdown on the malignant biological behaviors of OC cells.

### The effect of FOSL2 on cell apoptosis was reversed by Yvad-CMK

**Fig. 5:**
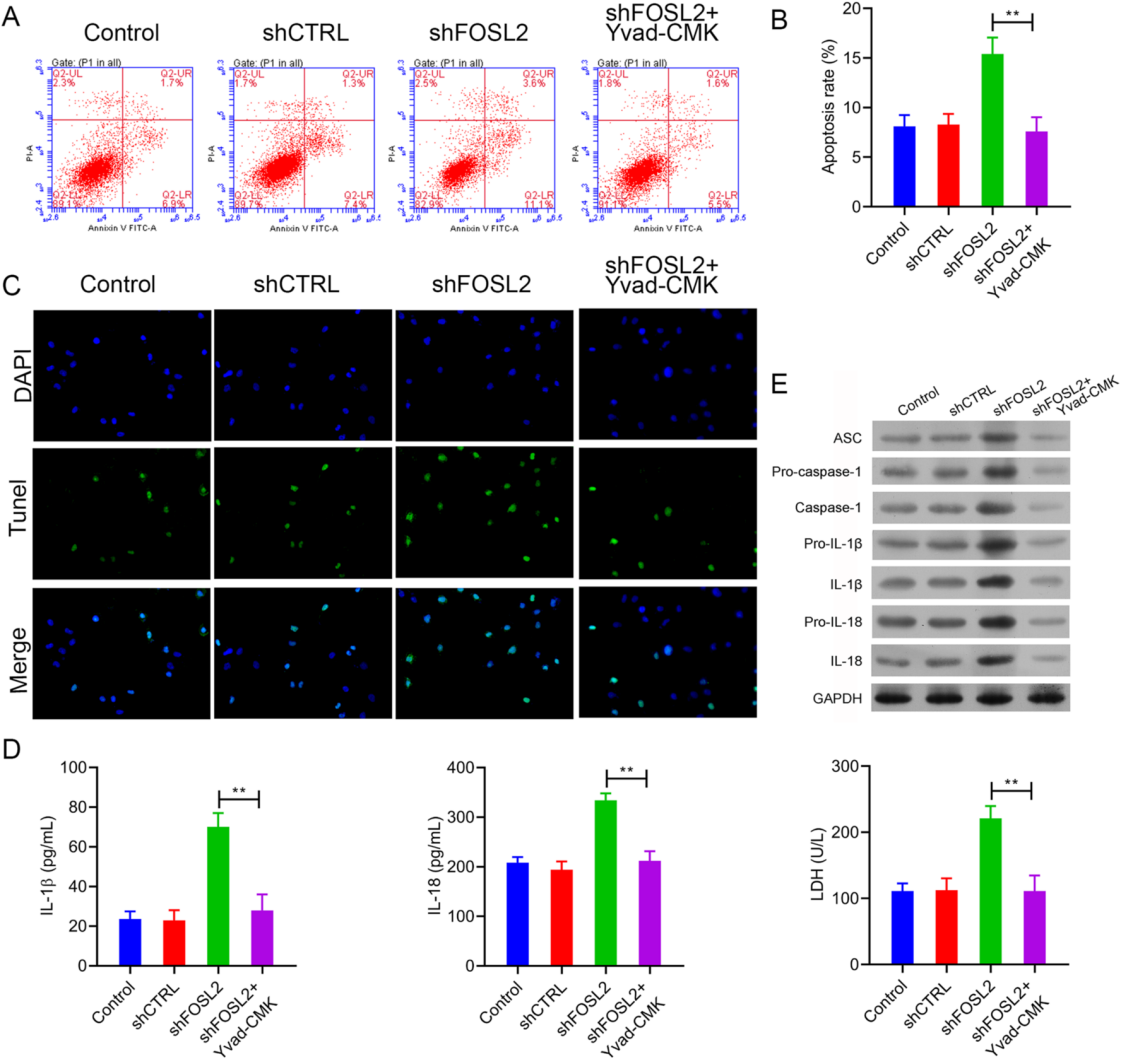
The effects of FOSL2 on cell apoptosis were notably reversed by Yvad-CMK. **A**, **B** The effect of FOSL2 and Yvad-CMK on cell apoptosis were detected by flow cytometry and TUNEL staining (**C**). **D** The secretion of IL-1β and IL-18 in cell supernatant were detected by ELISA assay, and the LDH activity in cell supernatant was detected by LDH release assay kit. **E** The expression of ASC, pro-caspase-1, caspase-1, pro-IL-1β, IL-1β, pro-IL-18 and IL-18 were analyzed by western blot. **P < 0.01 *vs*. shFOSL2 group

We determined the effect of Yvad-CMK on cell apoptosis induced by FOSL2 knockdown using flow cytometry. We found that FOSL2 knockdown increased cell apoptosis in A2780 cells, which was significantly mitigated by Yvad-CMK treatment (Fig. 5A–C). Moreover, after Yvad-CMK treatment, LDH activity due to FOSL2 knockdown in A2780 cells decreased (Fig. 5D, right panel).

FOSL2 knockdown upregulated the expression of ASC, pro-caspase-1, caspase-1, pro-IL-1β, IL-1β, pro-IL-18, and IL-18 (Fig. 5E). However, the expression of these proteins was reduced after Yvad-CMK treatment in FOSL2 knockdown A2780 cells (Fig. 5E). The IL-1β and IL-18 in A2780 cells detected via ELISA also showed similar results (Fig. 5D, left and middle panel). These findings demonstrated that inhibiting the activation of the inflammasome reverses the effect of FOSL2 knockdown on cell apoptosis.

## Discussion

Given that patients with OC often do not show specific symptoms in the early stage and no efficient clinical screening test is available, the diagnosis of OC remains challenging (Menon and Gentry-Maharaj 2018). Therefore, finding biomarkers for screening and molecular targeted therapy of this disease is important. In this study, we found that FOSL2 expression was significantly increased in OC tissues. Furthermore, it aggravated the malignant biological behavior of OC cells, which suggests that FOSL2 might be used as a new target for molecular therapy in OC treatment.

**Fig. 6:**
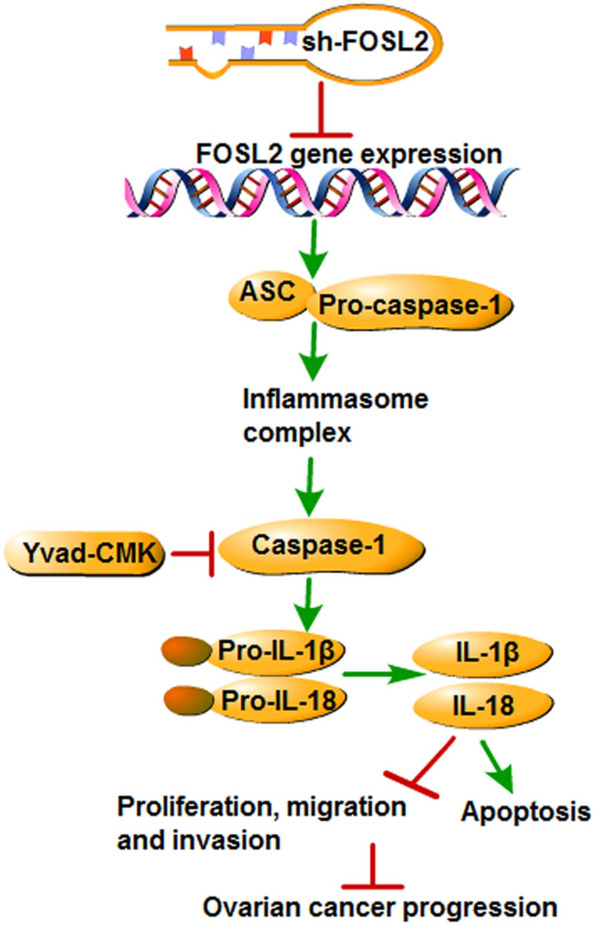
Schematic diagram of the proposed molecular mechanisms. A schematic diagram depicts molecular basis of the inhibition of FOSL2 aggravates the apoptosis of ovarian cancer cells through promoting the formation of inflammasome. Inhibition of FOSL2 could activate the inflammasome via up-regulation the expression of ASC, caspase-1, IL-1β and IL-18 to promote the apoptosis and suppress the proliferation, migration and invasion of ovarian cancer cells

With the advancement in immunological research and techniques, increasing evidence has shown that inflammasomes are associated with the occurrence and development of various tumors, including colorectal cancer, stomach cancer, melanoma, and lung cancer (Bo Hu et al. 2010; Deswaerte et al. 2018; Lasithiotaki et al. 2018; Zhai et al. 2017). Studying the effect of inflammasome on the development of malignant tumors is beneficial in terms of pointing the way forward for antitumor therapy. Inflammasomes are a family of multiprotein complexes in the cytoplasm that, when activated, can lead to the maturation and secretion of pro-inflammatory cytokines IL-1β and IL-18 (Schroder 2010). Therefore, they play an important role in the innate immune response. Our study found that silencing FOSL2 significantly increased the expression of caspase-1, IL-1β, and IL-18, which are characteristic markers of inflammasome expression (Schroder 2010). This indicates that the activation of inflammasome may be a possible approach for FOSL2 knockdown to regulate the development of OC. According to Schroder (2010), when cells are stimulated by DAMPs or PAMPs, NLRP3 autologously oligomerizes and assembles with ASC to recruit pro-caspase-1, triggering the inflammatory cascade (Schroder 2010). We also demonstrated that the expression of ASC was substantially increased when FOSL2 was knocked down, showing that FOSL2 regulated the progression of OC by mediating the inflammasome. This finding was further supported by inhibition of caspase-1 activity and the detection of cell functions and proteins expression in A2780 cells transfected with sh-FOSL2 and co-treated with Yvad-CMK. This indicates that FOSL2 knockdown inhibits cell proliferation, migration, and invasion and promotes the apoptosis of OC cells by activating the inflammasome (Fig. 6).

Cell apoptosis is a type of programmed cell death. Pezuk (2019) found that increasing the apoptosis level of tumor cells can effectively suppress tumor growth and proliferation (Pezuk 2019). Therefore, cell apoptosis could be a new approach to inhibit the growth and proliferation of tumor cells. Yang F et al. have reported that increasing the apoptotic level of OC cells can effectively inhibit the invasion, growth, and division of these cells, which may be related to the activation of α-NETA cytotoxic activity after apoptosis (Yang Fan et al. 2019). In this study, we found that inhibiting FOSL2 promoted the apoptosis of A2780 cells, which we were able to restore by Yvad-CMK treatment. This shows that FOSL2 may be used as a therapeutic target in combination with Yvad-CMK to treat OC.

LDH is a key enzyme in the anaerobic metabolic pathway that catalyzes the conversion of lactate to pyruvate with the reduction of NAD + to NADH and vice versa and serves as an important checkpoint of gluconeogenesis and DNA metabolism (Schumann et al. 2002). Many studies have shown an increased LDH activity in the cell supernatant due to extensive cell death that caused the release of intracellular LDH into the outside of the cytoplasm (Gallo et al. 2015). We also found that knocking down FOSL2 upregulated the LDH activity in the cellular supernatant of A2780 cells, but Yvad-CMK treatment suppressed it. This further confirms that FOSL2 regulated the cell apoptosis of OC by regulating caspase-1 expression.

In conclusion, our study showed that FOSL2 was highly expressed in OC tissues, and silencing FOSL2 significantly inhibited malignant cell development. This confirms that FOSL2 functions as an oncogene in OC development. However, the inhibitors of caspase-1 could reverse these effects, indicating that FOSL2 silencing plays a negative regulatory role in the progression of OC via mediating the activation of the inflammasome, which could be used as a novel therapeutic target.

## Data Availability

Notapplicable.

## Abbreviations

AP1: Activator protein-1
ASC: Apoptosis associated speck-like protein containing a CARD
Caspase-1: Cysteinyl aspartate specific proteinase 1
DAMP: Dang-associated molecular pattern
EMT: Epithelial-mesenchymal transitions
FOSL2: Fos-like antigen 2
HCC: Hepatocellular carcinoma
IL-1β: Interleukin-1β
IL-18: Interleukin-18
LDH: Lactate dehydrogenase
PAMP: Pathogen-associated molecular pattern
Pro-IL-1β: Interleukin-1β precursor
Pro-IL-18: Interleukin-18 precursor
PRRs: Pattern recognition receptors
